# A Layer-Arranged Meshless Method for the Simulation of Additive Manufacturing with Irregular Shapes

**DOI:** 10.3390/mi12060674

**Published:** 2021-06-09

**Authors:** Ming-Hsiao Lee, Wen-Hwa Chen, Ying Mao

**Affiliations:** 1 National Center for High-Performance Computing, National Applied Research Laboratories, Hsinchu 30076, Taiwan; 2 Department of Power Mechanical Engineering, National Tsing Hua University, Hsinchu 30013, Taiwan; whchen@pme.nthu.edu.tw (W.-H.C.); ymaoae@gmail.com (Y.M.)

**Keywords:** meshless method, additive manufacturing, 3D printing, layer-by-layer, layer-based

## Abstract

Additive manufacturing (3D Printing) has become a promising manufacturing method as it can produce parts in a flexible and efficient way, especially for very irregular parts. However, during the printing process, the material experiences a great temperature change from the melting temperature to room temperature; this causes high thermal strains and induces distinct deformations which degrade the quality of the printed parts, especially in metal 3D printing. In order to reduce possible problems and find possible solutions, a prior evaluation by simulation is often adopted. Nevertheless, since the 3D printing process generates parts in a layer-by-layer way, the analysis model should also be layer-by-layer arranged and used with a layer-by-layer based analysis process to simulate the layer-by-layer additive printing; otherwise, the simulation may not match the real behavior. In order to meet these requirements, a new meshless method is proposed to match the situations and handle these problems. As a meshless method, the modeling is not constrained by the element distribution. In addition, the analysis model generated with the proposed method can be arranged in a layer-by-layer way and combined with the proposed layer-by-layer analysis scheme, so it can then match and simulate the printing processes. Furthermore, the layer-by-layer arranged models can be automatically created, directly based on the STL (STereo-Lithography) geometry model, which is a de facto standard in the 3D printing industry. This makes the proposed approach more straightforward and efficient. To validate the proposed method, two parts with holes inside have been printed and simulated for comparison. The results show a good agreement. In addition, a highly irregular part has also been simulated to demonstrate the effectiveness and efficiency of this proposed method.

## 1. Introduction

3D printing is a new promising method to produce parts in many fields. It is because it can produce highly complicated parts more efficiently than traditional manufacturing methods, even for free-form-surface parts, while meeting the high accuracy requirement [1,2]. However, during using 3D printing to manufacture parts, some defects may arise, e.g., deformations due to the great thermal strains. In order to avoid these cases, a prior evaluation of the printing process to improve possible defects is preferred, especially through numerical simulations.

To simulate the problems about the deformations of the parts, the finite element method (FEM) is mostly adopted. However, the simulation of the 3D printing process has encountered difficulties due to the printing process of parts proceeds step by step and builds the result layer by layer. In order to simulate the process, the analysis method and model must be able to meet the layer-by-layer requirement. However, for irregular parts, the traditional mesh generators, e.g., using free-meshing, can only generate randomly distributed meshes which don’t meet the essential requirement. The mapped meshing also is not able to solve the difficulties. Obviously, the generation of layer-based analysis models is the bottleneck in 3D printing simulation for traditional simulation methods. Without layer-based models, the simulations cannot be performed. Therefore, even most of the main-stream software packages had encountered this problem and could not be used to solve this type of simulation until recently.

In the very early beginning, Zeng et al. [3] used an equivalent model to speed up thermal analysis for the 3D printing simulation. Liu et al. [4], Li et al. [5], and Michaleris [6] started with small-scale models which were regular and easy for mesh generation to simulate the local thermal and structural behaviors of the metal 3D printing. When the small and regular-shaped models are used, the mapped meshing technique can be adopted to generate layer-arranged element meshes for layer-by-layer analyses. However, this may not work for irregular-shaped models. Keller et al. [7,8] proposed a method which extracts residual strains from a small-scale model and then applies the residual strains to a large-scale model. They adopted the sliced geometry data used in the 3D printing industry to generate layer-based meshes for irregular-shaped parts and then did layer-by-layer analyses. Actually, the sliced geometry data, which is layer-by-layer and used by all 3D printing machines, also are generated based on STL geometry. It has proved to be an effective way for simulating 3D printing processes. In those studies, the three-dimensional layer-based meshes were generated on the basis of sliced data different from traditional FEM mesh generation. Livesu et al. [9] also used sliced geometric data to generate the mesh for the 3D printing simulation. In those methods, in order to generate a layer-based mesh, sliced data needs to be created first. However, the most common adopted geometric data in the 3D printing industry is STL formatted data. The sliced geometry data is not commonly used in CAD/CAE fields. Lee et al. [10], the same team of this study, proposes a layer-based mesh generation method directly based on the STL formatted data and a layer-by-layer simulation process to simulate the 3D printing simulation. This approach was demonstrated to be more straightforward and efficient.

Currently, in the 3D printing industry, geometry data in the STL format has been considered a de facto standard. This is because of its flexibility to represent irregular-shaped parts which are mostly encountered in the 3D printing industry. Geometry in STL format (or STL geometry) is different from traditional CAD geometries, which include all types of points, lines, surfaces and volumes. On the contrary, in STL geometry, the surfaces of three-dimensional parts are represented only with triangular facets. This has become a flexible and powerful way to represent three-dimensional irregular and free-form geometries and has been widely adopted in many application fields, such as computer-aided manufacturing, computer graphics and 3D printing. Currently, thanks to the rise of 3D printing, even the final parts including all types of geometric entities can be converted to STL geometry at one click with almost all main-stream CAD/CAE software tools. Moreover, in the 3D printing industry, during data transfer, most of the time only STL geometry is available. Because of these features, the proposed method adopts STL geometry to represent the geometry and the analysis domain. This can make the method easier and more straightforward to use. This also can lessen the complication of geometric data handling and transfer.

For the FEM-based method, layer-based mesh has been successfully developed and included by some main-stream commercial software packages, such as ANSYS, MSC/NASTRAN, in recent years.

**Figure 1:**
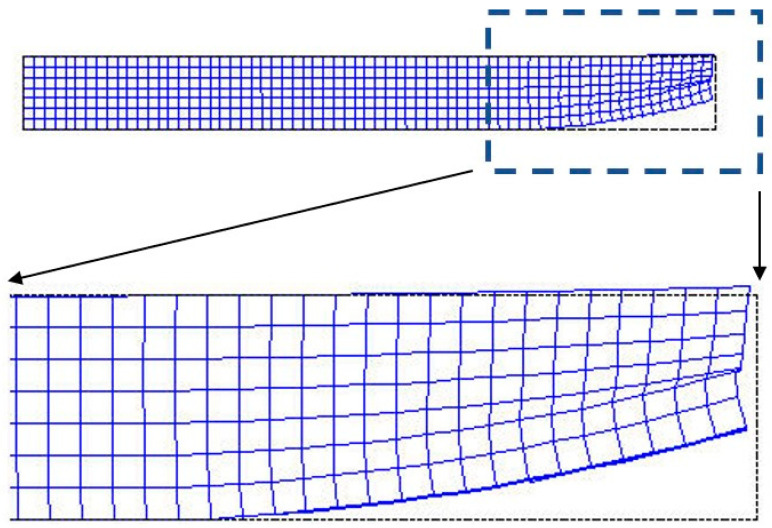
Extremely distortion of the mesh due to large deformation during a 3D printing process.

Although the FEM method is very powerful, it still has some intrinsic disadvantages due to the need of constructing element meshes; as such, the mesh generation is very tedious and time-consuming, the distortion of the elements could degrade the accuracy and cause the simulations to fail. For example, the layer meshes could become very distorted due to the large deformation in 3D printing cases, as shown in Figure 1.

On the contrary, the meshless (meshfree) method does not require any element meshes for the solution. This feature has become an advantage over the FEM. Because of this, based on the similar “meshless” idea, there have been emerging various meshless methods, such as, the element-free Galerkin method (EFGM) [11], the reproducing kernel particle method (RKPM), the h-p clouds method, and the meshless local Petrov–Galerkin method (MLPG), etc. These meshless methods have been applied to a variety of areas, especially in solid mechanics, such as two-dimensional sheets and shell structures [12], two-dimensional linear elastic fracture mechanics and crack growth problems [13], contact problems [14], three-dimensional structural problems [15,16,17], three-dimensional problems with complicated geometry [18], etc.

Although there were successful applications in many engineering fields, there still are few developed for 3D printing problems, basically because of the needs and the difficulties of the layer-by-layer models and simulation processes.

By the meshless method, Yaagoubi et al. [19] solved a thermal problem for the Selective Laser Sintering (SLS) process. Rodrigues et al. [20] using a meshless method to simulate 3D-printed specimens’ structural behaviors in standard tensile and compression tests. None of them simulates the 3D printing process. Mao et al. [21], on the same team of this paper, started to use a meshless method based on the EFGM to simulate the 3D printing process for regular-shaped objects. It demonstrated the effectiveness of the meshless method to do 3D printing simulations. However, the layer-based model for three-dimensional irregular-shaped parts was still a difficulty. Most meshless methods adopt background meshes [22], which can be used to define the analysis domain and serve for node generation; hence it is also needed to use free-meshing to generate the analysis models (i.e., extract the nodes out of the background meshes) for irregular-shaped objects. As the background meshes are randomly distributed, the node distribution is thus also randomly distributed. This type of node distribution also cannot meet the layer-by-layer requirement. Instead of using background meshes, Lee et al. [17], on the same team of this paper, proposed a different approach without using background meshes and directly used mathematical geometric entities to generate the analysis model. However, in the 3D printing manufacturing, mostly only STL geometry is available. Therefore, the current paper proposes a series of geometry-related schemes to create the layer-based model directly based on STL geometry and proposes a meshless-based method to simulate the 3D printing process for irregular-shaped objects. By this method, the status of the deformation during a layer-by-layer process can be predicted; thus, it can be used to evaluate and then improve part designs. In addition, in this study, the effect of some geometric features, such as holes, on the final maximum displacement has also been investigated.

## 2. Methods

As mentioned above, in order to simulate the layer-by-layer growing situation, the simulation should also be performed in a layer-by-layer process. To conduct that approach, a layer-based model is required. However, most meshless methods which adopt background meshes [22] use free-mesh generators to generate the models for irregular shaped objects, since the mesh are randomly distributed, so the node distribution is thus also randomly distributed. This type of node distribution cannot match the layer-by-layer requirement. Hence, a layer-by-layer simulation cannot be taken. Here a new layer-based meshless model generator is proposed. With this type of model, a layer-by-layer simulation process can then be conducted to simulate the 3D printing processes. Moreover, this layer-based model generator directly uses the STL geometry to produce the analysis model. This approach can fit the convention in the 3D printing industry, since STL geometry is the most common used geometry format.

### 2.1. Defining the Analysis Boundary and Domain

**Figure 2:**
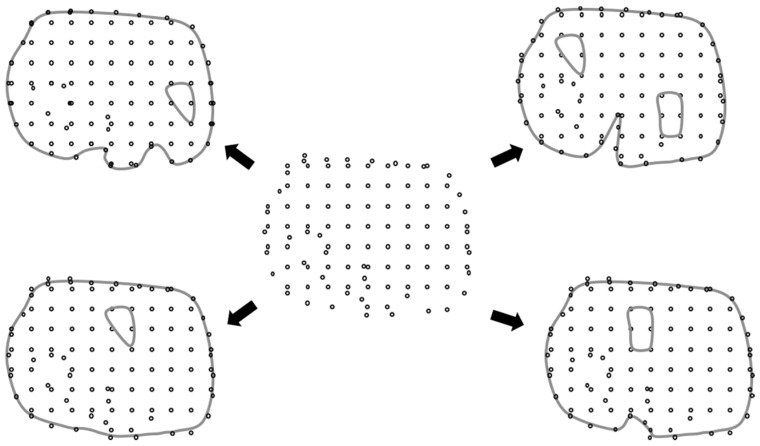
Ambiguity due to a model only with nodes.

In the finite element method, the analysis model itself provides enough information to define the domain and boundaries by the element mesh. The definition of analysis domain is not an issue in the finite element method. However, the meshless method does not use any elements, so the analysis models have only unconnected nodes which cannot provides adequate information to define the boundaries and analysis domain. For example, a set of nodes without extra information could generate ambiguity in representing the analysis domain as shown in Figure 2. Therefore, it is needed to employ additional mechanisms to uniquely determine the boundaries and analysis domains, especially for three-dimensional irregular objects. Some research takes advantage of the element mesh, called the background mesh, i.e., although the elements are not used to derive the shape functions, they are used to define the analysis domain and boundaries. However, for irregular parts, the background meshes used are generated by free-meshing, so the nodes in them are randomly distributed and cannot match the requirement of layer-by-layer arrangement in the 3D printing application. Here, a new method is proposed to generate the analysis model and well defines the analysis boundaries and domain without using the element mesh, moreover, the model can easily meet the layer-by-layer requirement, so that it can be used for layer-by-layer simulation. To achieve those features and functions, several new schemes are proposed.

### 2.2. Automatic Layer-Based Node Generator

**Figure 3:**
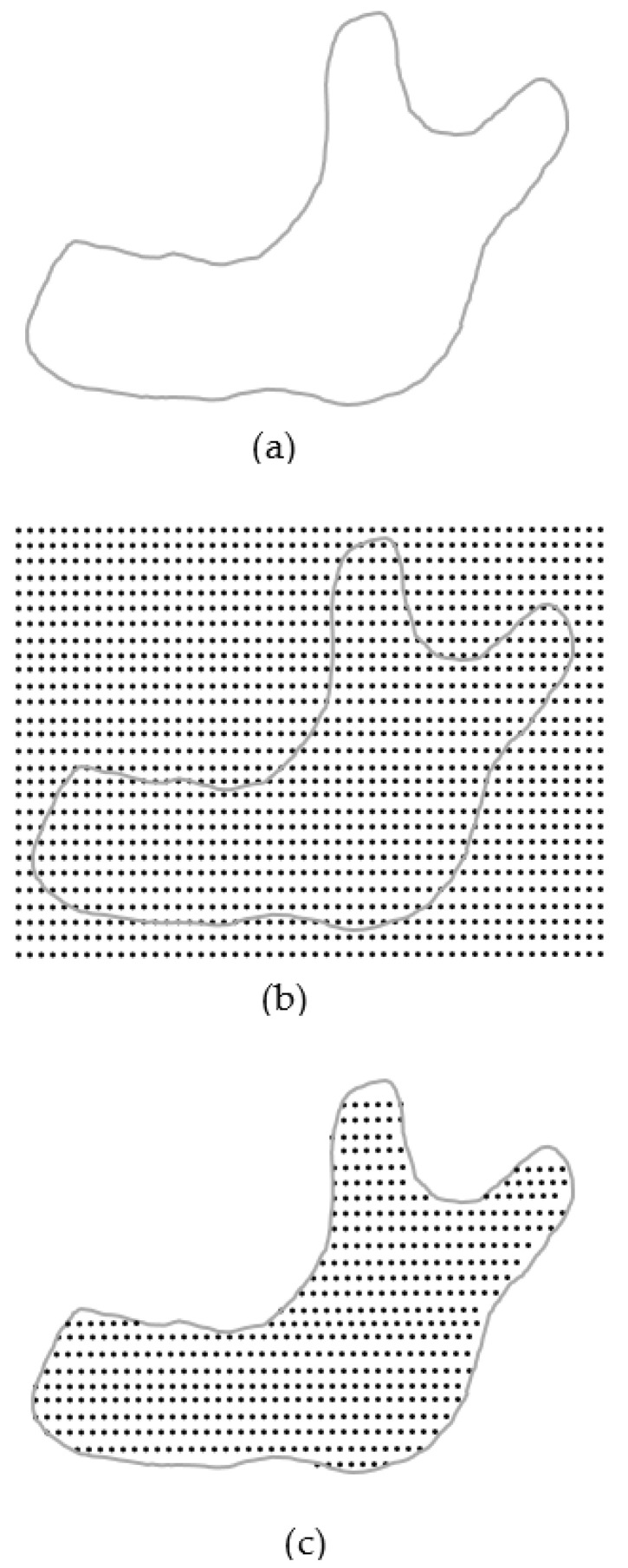
Layer-based node generator: (**a**) STL (STereo-Lithography) geometry; (**b**) initial nodes; (**c**) final nodes.

The main entities to define the analysis model are nodes in a meshless method. Nodes represent points on which the interpolation to derive the variable’s value at certain position in the analysis domain is based. Once the analysis domain is determined, i.e., with the STL geometry, the nodes can then be generated accordingly, distributed inside the analysis domain. In order to match the layer-by-layer printing situation, a layer-by-layer arranged nodal model is needed. This type of nodal models can be generated in the following procedure, as shown in Figure 3,

(1) Import the geometry data in STL format, Figure 3a
(2) Pave nodes regularly, layer by layer, over the entire analysis domain, Figure 3b
(3) Delete those nodes outside the analysis domain and keep the nodes inside the domain for each layer, Figure 3c. The method to judge if a node is inside or outside the analysis domain is explained later.

In this, the nodes are regularly paved in the beginning and then filtered to leave the inside nodes. These operations can be done automatically with the proposed schemes.

### 2.3. Inside/Outside Checking

**Figure 4:**
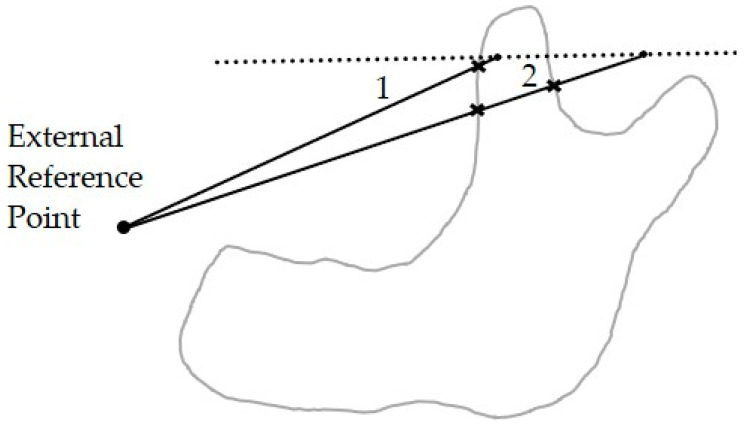
Inside/outside checking.

For the node generation scheme mentioned above, the nodes are paved regularly first to cover the entire analysis domain even in three-dimensional space; however, some of the nodes are outside the analysis domain and needed to be excluded. Therefore, it is necessary to determine if certain nodes are inside or outside the analysis domain. The proposed mechanism to check if a point is inside or outside the analysis domain is shown in Figure 4. First, an external reference point, i.e., located outside the analysis domain, is created. Second, connect the external reference point and the discussed point to form a line. Third, check how many times the line crosses the boundary. The criteria are if the connecting line crosses the boundaries an odd number of times, e.g., line 1 crosses the boundary once, then the discussed point lies within the analysis domain; otherwise, the point is outside the analysis domain, e.g., line 2 crosses twice.

With this mechanism, one can effectively determine if the discussed node is inside the analysis domain or not, even when there are internal holes or complicated concave boundaries contained in a complicated three-dimensional analysis domain.

#### 2.3.1. Forming Bounding Box

**Figure 5:**
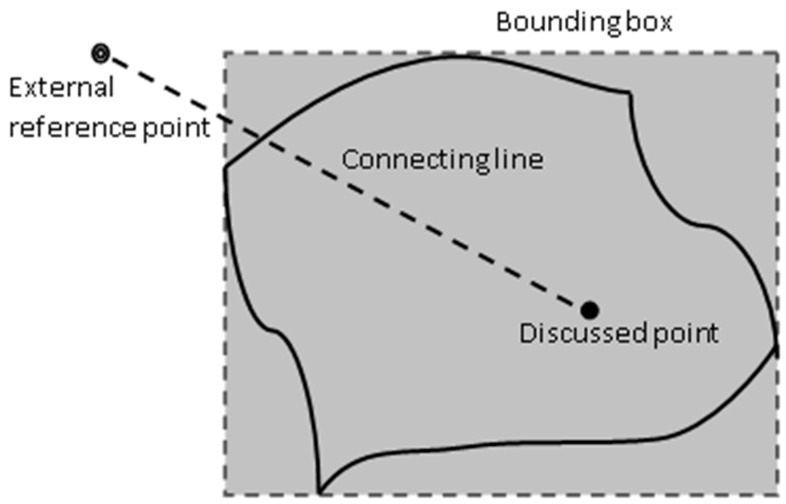
Bounding box.

In order to assure the created reference point is outside the analysis domain. A bounding box is first formed. This bounding box should be big enough to cover the entire analysis domain, as shown in Figure 5. This can be fulfilled by finding the minimum and maximum coordinates, in x, y, and z directions respectively, from all the boundary nodes, i.e., the vertex nodes of the STL’s triangular facets. Then, create an external reference point outside the bounding box, which is assured to be outside the analysis domain.

After the reference point is defined, then, connect it to the discussed point with a connecting line and then the procedure to count the number of times the connecting line crosses the boundaries can be performed.

#### 2.3.2. Crossing Boundaries

**Figure 6:**
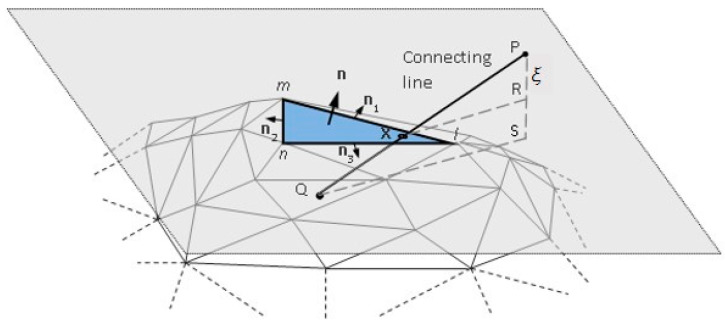
Crossing boundary check.

For three-dimensional objects, since the boundary surfaces are formed by triangular facets, so in order to determine if a connecting line crosses the surface boundaries of a three-dimensional analysis domain, it can be done by checking if the connecting line intersects any triangular facets. In other words, when one wants to know how many times a connecting line crosses the surface boundaries, one can just check how many triangular facets have been intersected by that connecting line. To check the intersection of a connecting line and a triangular facet, the following mechanism is proposed. First, as shown in Figure 6, assume a triangular facet is being checked. a connecting line may intersect the unbounded plane on which the discussed triangular facet lies at point x. If the line intersects the triangular facet, point x should be within the facet; otherwise, if the point x is outside the discussed facet, then the line does not intersect that facet.

To conduct this, first, the point x where the line intersects the plane needs to be found. To do this, as illustrated in Figure 6, first, compute the normal distance from point P to point R on the plane and P-S, which is the component of P-Q in the normal direction of the plane. *ξ* is the length ratio of P-R to P-S. One can then determine the coordinates of the intersection point x, u(x), with the coordinates of point P, u(P), and Q, u(Q), by linear interpolation:

u(x) = (1 − *ξ*) u(P) + *ξ* u(Q) (1)

As shown in Figure 6, there are three outward normal vectors to the three edges of that facet, namely n_1_, n_2_ and n_3_. To determine if point x is inside an edge, connect a vertex of the discussed edge and point x to form a vector, e.g., l–x, and take a scalar product between vector l–x and the normal vector to that edge, e.g., n_1_ or n_3_. If the result is negative, point x is behind that edge. If point x is behind all three edges, it means that the point x is inside that facet and the connecting line has intersected that facet. Similarly, continue to check other facets in the same way and then the total number of times can be obtained.

### 2.4. Formulation of Meshless Method for 3D Printing

Once the layer-based analysis model is ready, in which the nodes are layer-by-layer arranged, the solution can be performed. To obtain the solution of a 3D printing problem, the formulation for the proposed meshless method is adapted from the EFGM [11]; however, some different approaches are also adopted. It can be derived as follows.

**Figure 7:**
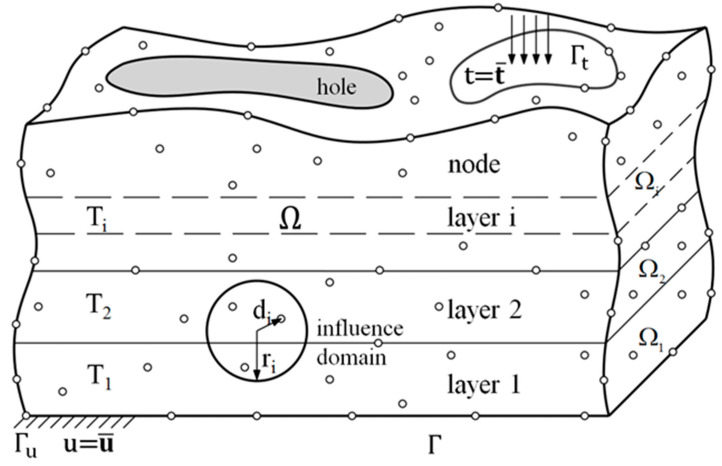
The meshless method for layer-by-layer analysis.

As depicted in Figure 7, consider a three-dimensional structure Ω which consists of *n* layers, Ω_1_, Ω_2_, … Ω_n_. The domain Ω is enclosed by the boundary Γ.

For an isotropic elastic problem dealing with a 3D printing case, the total potential energy of the system subjected to a uniform temperature change ΔT is

(2) $$\Pi =\frac{1}{2}\int_{\Omega_{1}+\Omega_{2}+\ldots \Omega_{n}}\varepsilon^{T}E\varepsilon d\Omega -\int_{\Omega_{1}+\Omega_{2}+\ldots \Omega_{n}}\varepsilon^{T}{E\varepsilon }^{\mathrm{th}}$$

where **ε** is the strain vector, **E** is the material matrix and **ε**^th^ is the thermal strain vector induced by the temperature change ΔT, i.e.,

(3) $$\varepsilon^{\mathrm{th}}=\alpha \Delta T$$

where **α** is the thermal expansion coefficient.

The displacement **u** at any position can be interpolated from the global nodal displacement **D** by the global shape function **Φ** as

(4) u=ΦD

The strain **ε** in Equation (2) can be derived from displacement **D** and the gradient matrix **B**, i.e.,

(5) ε=BD

Thus, the total potential energy **Π** in Equation (2) becomes

(6) $$\Pi =\frac{1}{2}D_{}^{T}KD-D_{}^{T}F$$

where

(7) $$K=\int_{\Omega_{1}+\Omega_{2}+\ldots \Omega_{n}}B^{T}EBd\Omega$$

and

(8) $$F=\int_{\Omega_{1}+\Omega_{2}+\ldots \Omega_{n}}B^{T}{E\varepsilon }^{\mathrm{th}}d\Omega$$

In the above, **K** denotes the global stiffness matrix and **F** is the corresponding global force vector. Then the equilibrium equation can be obtained by taking the minimum of the potential energy as

**KD** = **F** (9)

Then the global nodal displacement **D** can be derived by solving this equation.

### 2.5. Automatic Layer-Based Quadrature Scheme

To form the integrals in the above equilibrium equation, one needs to use a proper quadrature scheme. Integration points actually represent the real volumes of the material at certain location. In 3D printing, the material is deposited evenly layer by layer; in order to catch the evenly growing behavior of the deposited material, since the layer thickness is uniform, the integrals of the **K** matrix and the **F** matrix should also increase uniformly, layer-by-layer. Hence, a uniformly layer-by-layer arranged integration point scheme is needed to match the uniform layer-by-layer deposition.

**Figure 8:**
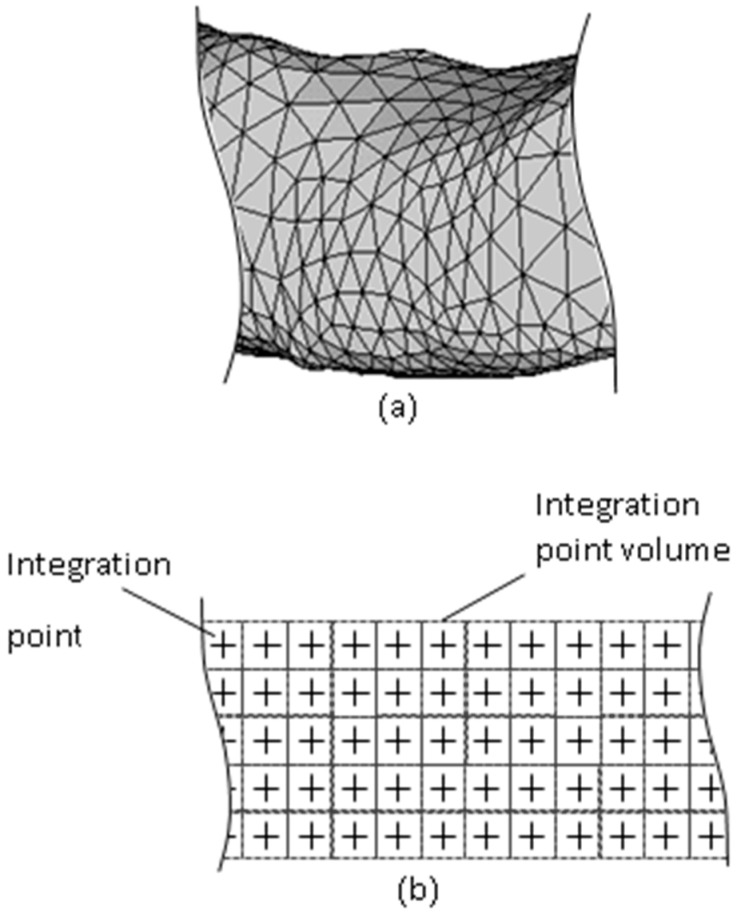
(**a**) Other meshless methods with a background mesh and (**b**) the proposed layer-based integration point scheme.

Traditionally, for numerical spatial integration, the Gaussian quadrature scheme is often adopted, even by most meshless methods [11]. However, the integration points in the Gaussian quadrature scheme are not uniformly distributed, so this cannot match the uniform layer-by-layer printing in which the same thickness of material is deposited for each layer. Moreover, if a meshless method adopts a randomly distributed background element mesh for irregular parts, as shown in Figure 8a, since the mesh decides the distribution of the integration points, the distribution of the integration points is also randomly distributed, and cannot properly match the uniformly layer-by-layer growing situation.

Herein, a layer-based quadrature scheme is proposed. In this, the background element mesh is not needed and the integration points are uniformly paved layer by layer, as shown in Figure 8b. Then, the integrals of the **K** and **F** matrices can increase accordingly. Moreover, the process is automatically performed in this scheme, as described below.

**Figure 9:**
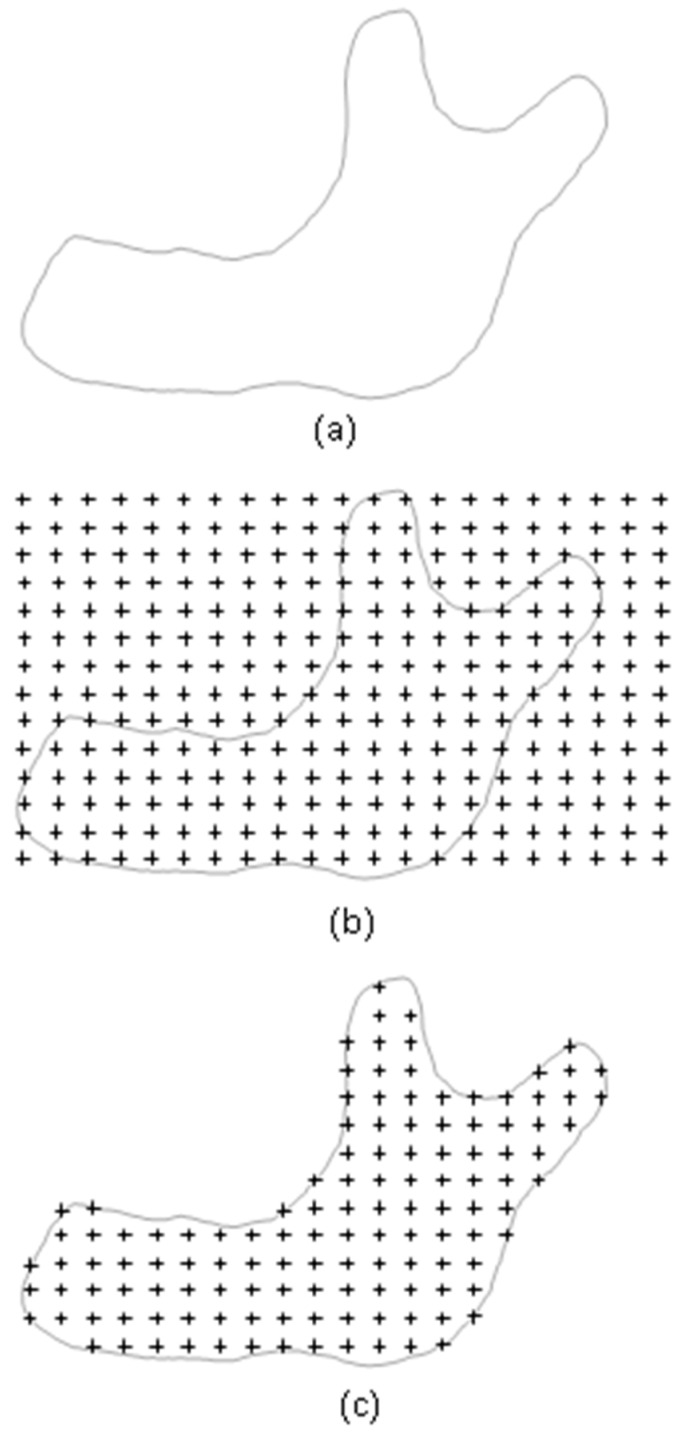
Generation process of the uniform layer-based integration point scheme: (**a**) STL geometry; (**b**) initial integration points; (**c**) final integration points.

These types of integration points can be generated in the following procedure, as shown in Figure 9,

(1) Similar to the generation of nodes described above, take advantage of the same geometry data in STL format, Figure 9a
(2) Pave integration points layer by layer over the entire analysis domain, Figure 9b
(3) Delete those integration points outside the analysis domain and keep the inside ones. for each layer, Figure 9c. The method to judge if an integration point is inside or outside the analysis domain is the same as the one used for node generation.

Then, the integrals in above equations can be calculated by the following equation,

(10) $$\int_{\Omega }fd\Omega =\sum_{i}^{n_{p}}\sum_{j}^{n_{q}}\sum_{k}^{n_{r}}\alpha_{i}\beta_{j}\gamma_{k}f_{ijk}$$

where *f* is any function, and *n_p_*, *n_q_*, *n_r_* are the numbers of integration points along three axis directions. *f_ijk_* is the value of the function *f* at a particular integration point. *α_i_*, *α_j_* and *α_k_* represent the weights of certain integration points in three axis directions. Here, they all are the same for each integration point in this uniform integration point scheme. Ω denotes the whole volume and analysis domain.

### 2.6. Layer-by-Layer Growing Process through Reduced K

**Figure 10:**
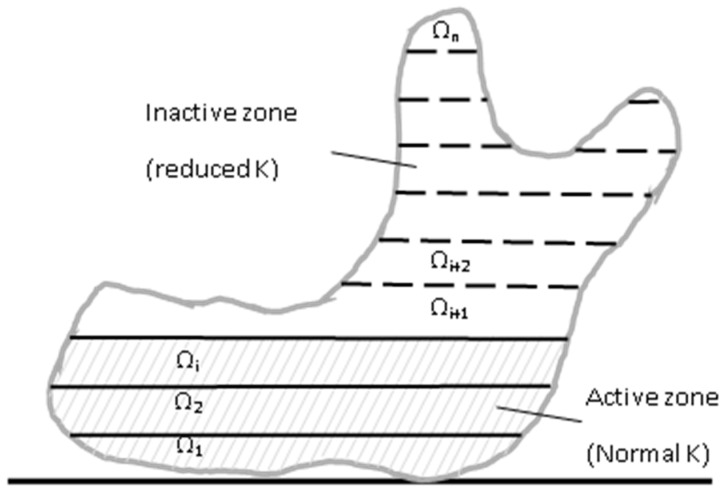
Layer-by-layer printing through reduced K.

In the proposed quadrature scheme, each integration point is independent, i.e., not attached to any elements or cells. As an integration point represents a certain real volume of the material, the material for each independent integration point can be different. This is an important feature for dealing with the layer-by-layer growing process. Since during the printing process, as the printing material is deposited increasingly, some part of the material is already present (i.e., active), but the other portion is not yet present (i.e., inactive), as shown in Figure 10; however, the analysis domain on which the global **K** matrix is based is the same. In this situation, a way to take the inactive portion into account is to change the material properties of those integration points in that “inactive” portion, i.e., reducing the Young’s modulus to a nearly negligible value. The local and global **K** matrix will be reduced accordingly to reflect the layer-by-layer growing behavior. In other words, when the printing is printing up to the ith layer, to obtain the domain integration for the global **K** matrix, only Ω_1_, Ω_2_, … Ω_i_ are effective and Ω_i+1_, Ω_i+2_, … Ω_n_ are near zero.

### 2.7. Layer-by-Layer Simulation Procedure

**Figure 11:**
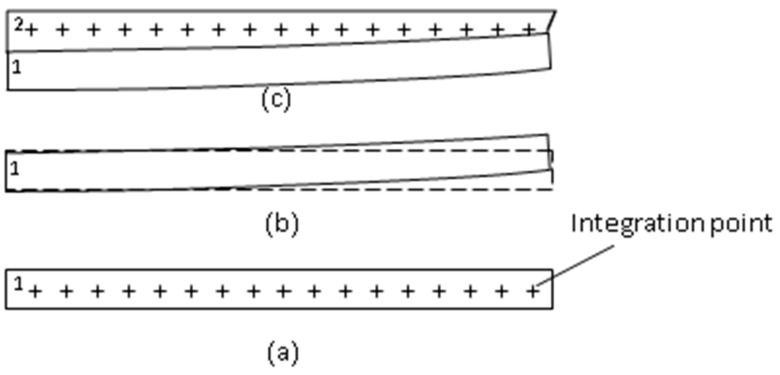
Layer-by-layer printing process simulation: (**a**) pave one layer; (**b**) deformed; (**c**) pave another layer.

After the nodal model and the layer-by-layer arranged integration points are ready, a layer-by-layer analysis procedure can be performed with it. In the beginning, first deactivate all the integration points, and then activate those integration points in certain layers one by one in order, i.e., in each analysis step. Activating a layer (of integration points) is just like newly-paved material layer in the real printing process. The layer-by-layer analysis procedure is as follows:

(1) Generate the nodal model and integration points layer by layer
(2) Deactivate all integration points, first
(3) Pave one layer of volume (material) by activating those integration points which represent that layer, e.g., the layer labeled 1 shown in Figure 11a
(4) Apply a temperature drop to the newly-activated layer, i.e., a thermal loading
(5) Calculate the **K** and **F** matrices for each integration point. However, the **K** matrices of those integration points which are not in the active layers are reduced by reducing the Young’s modulus by one millionth, i.e., nearly no stiffness for those integration points (volumes).
(6) Assemble all integration points’ **K** and **F** matrices to form the global **K** and **F** matrices
(7) Apply the boundary conditions, e.g., portion or the whole of the bottom is fixed.
(8) Perform a structural analysis step, in which the temperature cools down to certain temperature, e.g., room temperature 25 °C or others
(9) Solve the equilibrium equations to obtain the deformation and displacements caused by thermal shrinkage. The deformation is schematically shown in Figure 11b, where the dashed line denotes the original shape and the solid line denotes the deformed shape.
(10) Repeat steps (3)–(9) to activate the next layer, e.g., the layer labeled 2 shown in Figure 11c, and do the next analysis step.

As the number of real printing layers is rather large and the layers are thin, in order to avoid numerical calculation difficulties encountered in dealing with highly thin structures and to speed up the simulation, simplification is adopted by including several real material layers in each analysis layer (step), called the effective layer thickness. This can save distinct computing time; therefore, the effect of the effective layer thickness will be investigated below to obtain a reasonable thickness which can be used to get acceptable results.

## 3. Validation

**Figure 12:**
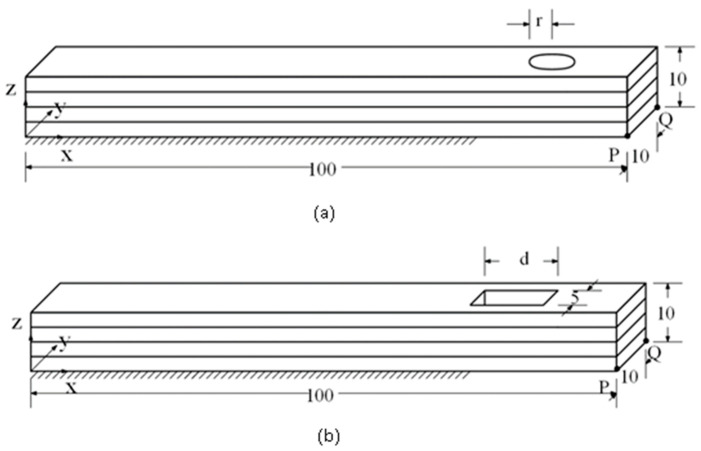
Models and setups for the validation case (for both simulation and experiment): (**a**) bar with circular hole and (**b**) bar with rectangular hole.

To validate the proposed schemes, a comparison between simulations and experiments was conducted. Two long bars with holes, circular or rectangular, were printed and simulated, subjected to the same conditions. The dimensions and shapes of the parts are shown in Figure 12. Since the bars have holes in them, they are no longer regular parts. The sizes of the long bars are 100 × 10 × 10 mm^3^. The radius of the circular hole (r) is 2.5 mm. The size of the rectangular hole is 10 (d) × 5 mm^2^. It was printed with a Fused Deposition Modeling (FDM) 3D printer (UP Box Plus 3D Printer, Tiertime). The material used was Polylactic Acid (PLA, Modex, Taiwan). For the printing process, 3/4 of the bottom of the long bar is fixed to the base table and another 1/4 is set free of constraint as the boundary condition for simulation and printing setup. This setup is to simulate the frequent failure in real printings in which a long object’s end portion often detaches from the base table due to the induced thermal stains during the 3D printing process. If the printing continues after the partial detachment, the printed part will deform visibly. This is helpful and easier for evaluating the relationship between the thermal strains and deformation during the printing.

### 3.1. Experiment Setup

For the experiment setup, the same team of this study had previously conducted related measurements and experiments to solve regular-shaped cases [21]. Some obtained setting data and material data are also adopted in this study.

**Figure 13:**
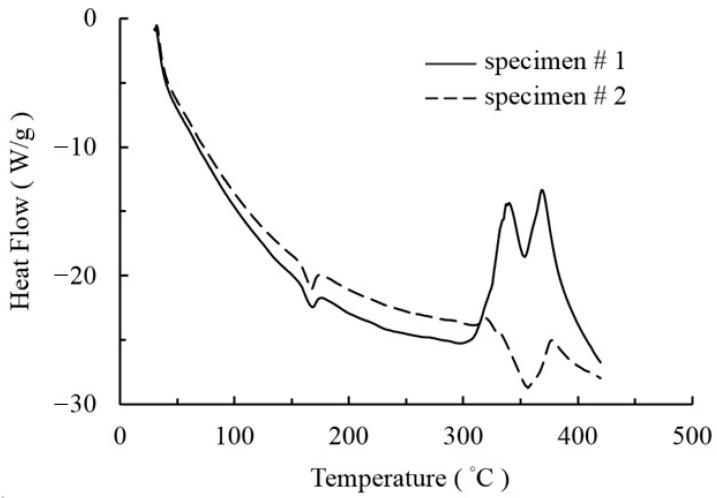
Differential Scanning Calorimeter (DSC) curves of Polylactic Acid (PLA) specimens.

The temperature change, i.e., from the melting temperature to room temperature, is the only loading for this case. The melting temperature is critically important. In order to obtain an accurate melting temperature, a DSC (Differential Scanning Calorimeter) experiment was conducted, as shown in Figure 13; as a result, the melting temperature (T_m_) of the PLA material used in the experiments is 158.2 °C. In addition, the Young’s modulus of the used PLA material is also tested with the printed specimens and the value is 583.40 Mpa. The material properties obtained from the experiments are used for the simulations.

**Figure 14:**
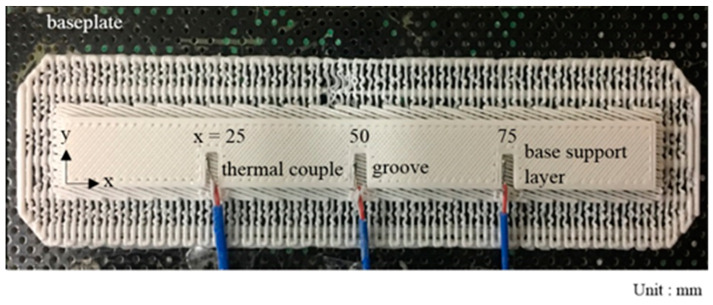
Distribution of three thermocouples.

The temperature for each layer varies during layer-by-layer printing. When one layer is just printed, it will cool down quickly; however, before it reaches room temperature, the next hot layer will be deposited and experience a similar cooling down process. This process continues layer by layer, in order. Hence, at a certain time, the temperatures in different layers are different, but they experience a similar cool down process, in order. To know the layer-by-layer temperature history for each layer during the printing process, the layer temperature measurement was conducted by using three T-type thermocouples put on the base support layer. They are distributed evenly as shown in Figure 14. From the experiment data, the widest temperature variation in three thermocouples in a layer is about 5.2 °C during the printing process. It is considered small compared with the total temperature drop; hence, it is adopted to assume the temperature of each layer to be uniform during printing.

**Figure 15:**
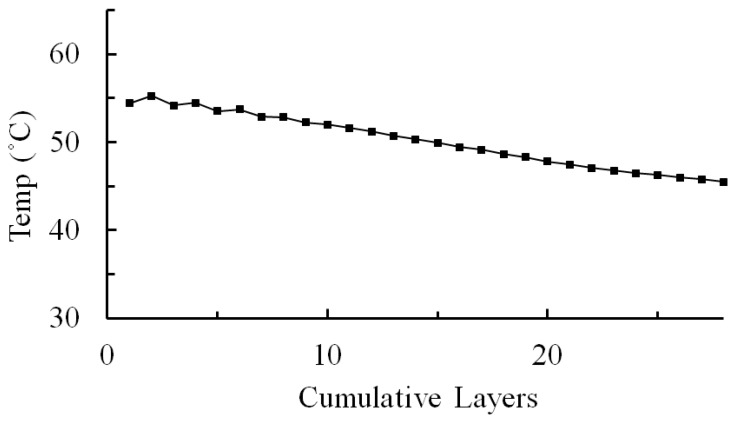
Average temperature history during the printing process.

The average temperature history derived from three thermocouples, i.e., T_1_, T_2_ and T_3_, in layer-by-layer wise during the printing process is shown in Figure 15. It shows that the temperature of a layer experiences a sudden temperature drop right after being deposited, then stays around 40~50 °C until the end of the printing, and then the whole part cools down to room temperature. It is also assumed that other layers will experience the same temperature history during the layer-by-layer printing. Hence, the temperature history of each layer was used for the simulations, as shown in Table 1.

### 3.2. Simulation

To simulate the test cases, the boundary conditions and loading are the same as those used for the experiments. The Young’s modulus is 583.40 MPa, Poisson’s ratio is 0.35, the thermal expansion coefficient is 8.5 × 10^−5^ mm/mm °C and the melting temperature is 158.2 °C [21]. For the boundary conditions, ¾ of the bottom of the long bar is fixed to the base table and another ¼ is free of constraint. The only loading in these cases is thermal loading, i.e., the material of each layer experiences temperature change from melting point to room temperature after being deposited. In that, the temperature changes are as described above. To apply the thermal loading layer by layer, the temperature change for each layer is shown in Table 1. After all printing is finished, the temperature of the whole part drops to room temperature.

### 3.3. Effective Layer Thickness

As the number of real printing layers is rather large and the layers are thin, this could cause numerical calculation difficulties and consume a large amount of computing time. Simplification by using the “effective layer thickness”, which includes adopting several real material layers in each analysis step. In order to choose a reasonable effective thickness which can still obtain acceptable results, various effective layer thicknesses were used to simulate the same case. To conduct this, real PLA printings with a thickness of 0.1, 0.2, or 0.3 mm are performed. Corresponding simulations for the case of a long bars, 100 × 10 × 10 mm^3^, without holes were conducted with different effective layer thicknesses, from 1.43 to 5 mm. The maximum displacements and deviations (compared with the real printing) for different effective thickness are shown in Table 2, Table 3 and Table 4. The results show that, for all cases, an effective thickness of 1.43 mm is the most accurate one. Therefore, an effective thickness around 1.5 mm can be used for the simulation to obtain acceptable results. Further simulations will adopt this effective layer thickness of 1.5 mm.

### 3.4. Result Comparison for the Validation Cases

**Figure 16:**
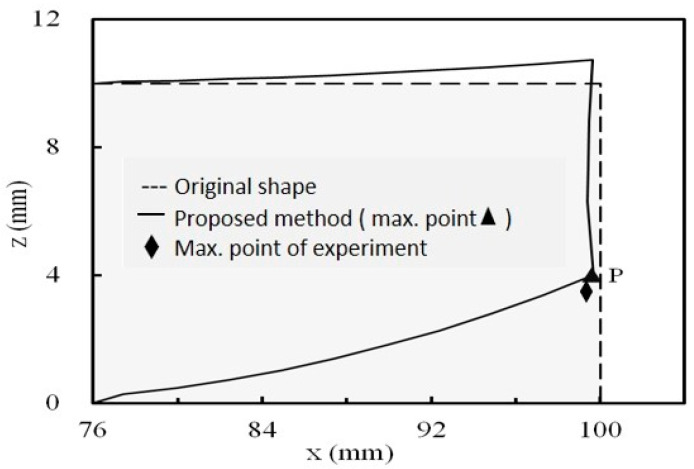
Displacements and deformation at the longitudinal end for circular hole case.

**Figure 17:**
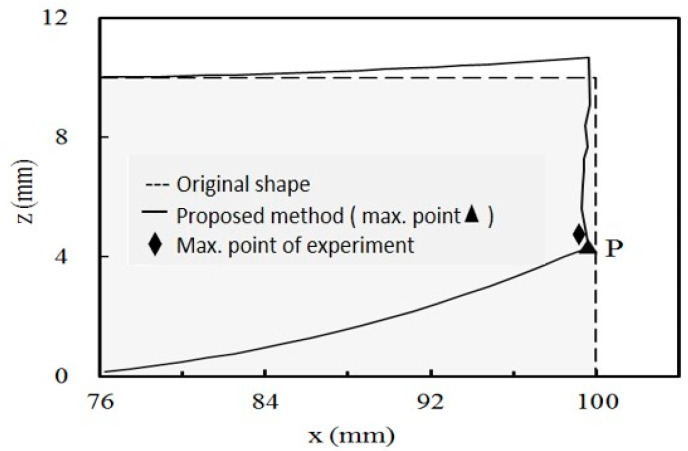
Displacements and deformation at the longitudinal end for rectangular hole case.

To compare the results from the experiments and simulations, in the case of a printing layer thickness of 0.2 mm, the displacements and deformation shape of the simulation of the one with a circular hole are shown in Figure 16 where the maximum displacement of the experiment is also included to show the comparison. The results from both show a good agreement, as shown in Table 5. The results of the case with a rectangular hole are shown in Figure 17. They also show a good agreement, as shown in Table 5. As compared with the FEM-based method, partly developed by the same team [10], the results also show a good agreement, as shown in Table 5, but it is difficult to say which method is better. These results validate the effectiveness of the proposed method.

According to the results, the bar bends, in both cases, at the longitudinal end as shown in Figure 16 and Figure 17. In addition, the layer-by-layer effects can be observed at the end edge. It is that, since the lower layers deformed before the upper layers were deposited, the end edge is no longer straight. The results show that the layer-by-layer effect causes a complicated deformation shape at the bar’s end.

After being validated, then the method can be used to investigate other cases.

Furthermore, the proposed method was used to investigate the effect of the sizes and positions of the holes on the final deformations. For the case with a circular hole, the radius of the circular hole was investigated. It is found that the maximum displacements increase as the radius increases, as shown in Table 6. It seems that the bigger holes reduce the stiffness to contain the thermal strains. The experiments also show the same trend. If the radius is fixed, i.e., 2.5 mm, and the position varies, it is found that the maximum displacements decrease as the hole moves closer to the end of the bar, as shown in Table 7. The experiments also show the same trend. However, the influence is very little. With these cases, the simulation results are in agreement with those of experiments. This validates the proposed method again.

**Figure 18:**
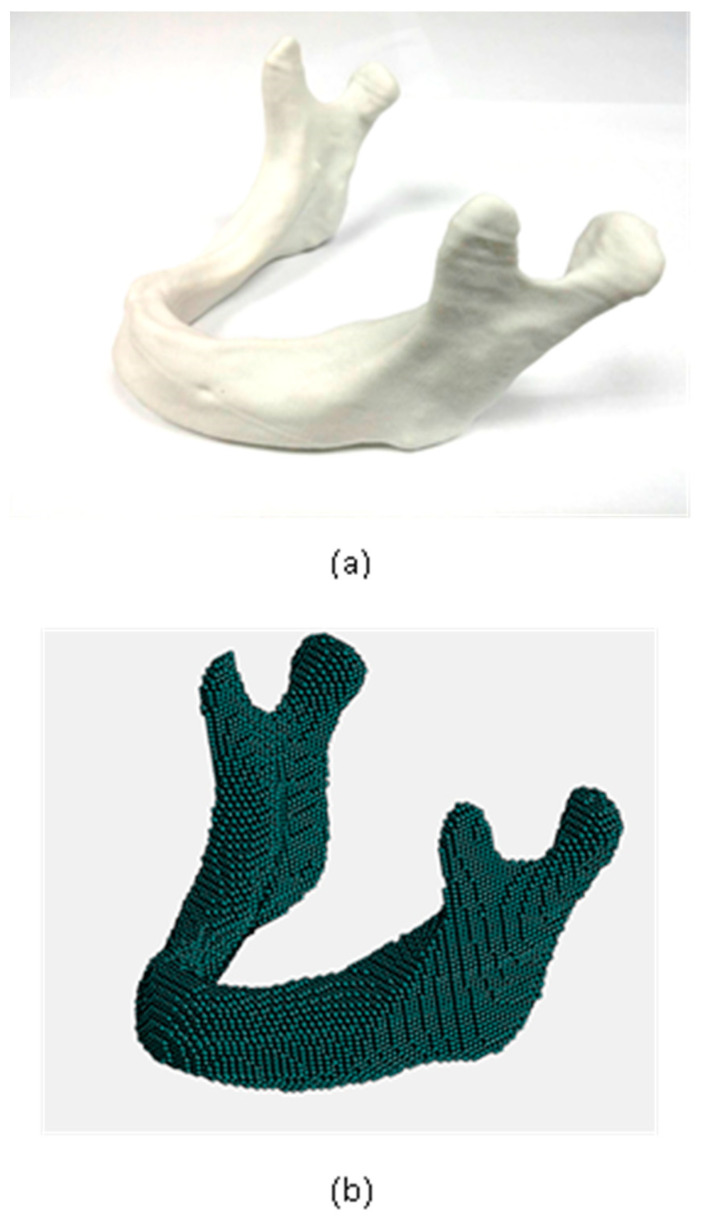
Geometry and meshless model of a mandible part: (**a**) real part (**b**) meshless model.

To demonstrate the capability of the proposed method in handling irregular-shaped objects, the proposed method was adopted to simulate a much more complicated case. This is to simulate a mandible part printed with metal 3D printing. The shape of the specimen is extremely irregular and free-form, as shown in Figure 18a. The geometric data is from a STL geometry. The meshless model can be efficiently and automatically generated with the proposed method, as shown in Figure 18b. The material tested here is titanium. Since the thermal strains of the metal are much larger, the deformation could be more serious. Significant deformation will degrade the accuracy of the parts in size. A prior simulation is even more needed.

**Figure 19:**
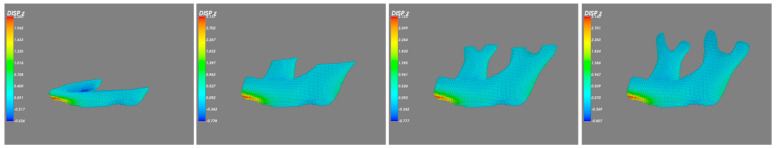
Displacement results during the printing solved by the meshless method.

The Young’s modulus is 120 GPa, Poisson’s ratio is 0.3, thermal expansion coefficient is 8.2 × 10^−6^ mm/mm °C and the melting temperature is 1650 °C. To simulate this case, the bottom is fixed to the base plate. During the printing process, the temperature of the material cools down from the melting temperature to room temperature 25 °C. The simulation stages and results are shown in Figure 19. It is found that the specimen deforms the most at the edge. The maximum displacement at that end is 3.14 mm.

**Figure 20:**
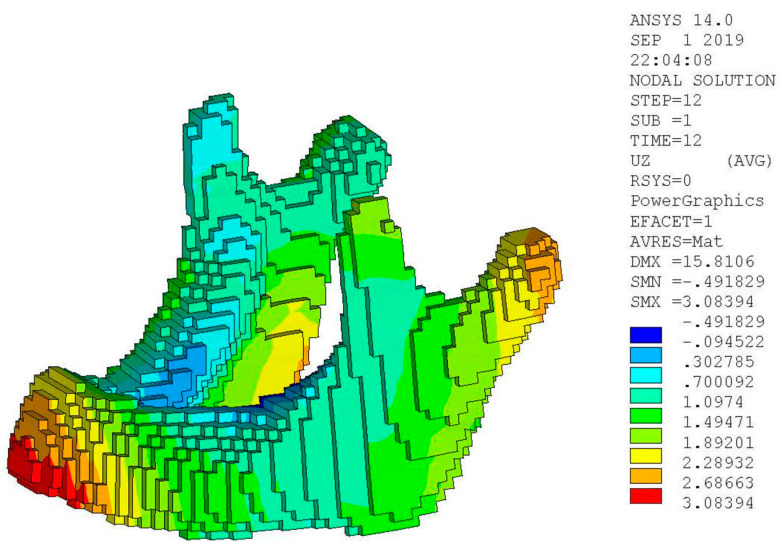
Displacement results solved by the layer-based FEM method [10].

In addition to the simulation with the proposed meshless method, a FEM simulation with the layer-based mesh generator and process proposed by Lee [10] was also conducted for comparison, as shown in Figure 20. The maximum displacement by the FEM method is 3.08 mm. The results from both methods show a good agreement, as shown in Table 8.

This case demonstrates that the proposed method can handle objects with very complicated irregular shapes.

**Figure 21:**
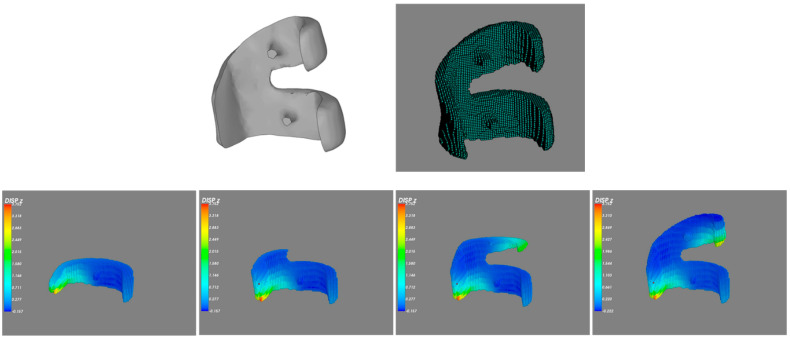
Model and displacement results during the printing solved by the meshless-based method.

The next case is also a free-form-surface part—a part of an artificial joint. The shape of the part is shown in Figure 21. The geometric data is from a STL formatted geometry. The meshless model is also shown in Figure 21. The material tested here is titanium as in the above case. The simulation results with the proposed meshless-base method is also shown in Figure 21.

**Figure 22:**
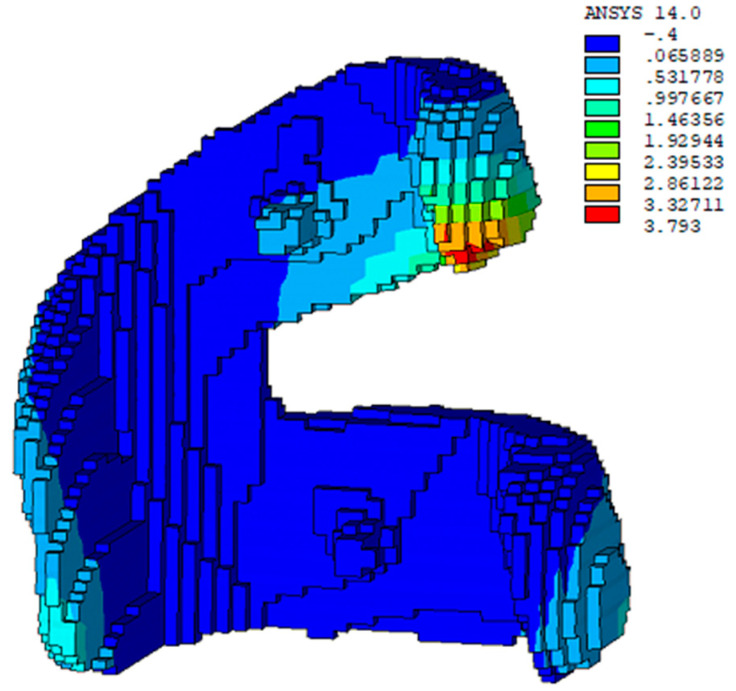
Displacement results solved by the layer-based finite element method (FEM) method.

In addition to the simulation with the proposed meshless-based method, a simulation with the layer-based FEM method was also conducted for comparison, as shown in Figure 22. The maximum displacement by the FEM-based method is 3.793 mm and the one by the meshless-based method is 3.752 mm. The results from both methods also show a good agreement, as shown in Table 9.

## 4. Discussion and Conclusions

3D Printing is a potential manufacturing method, especially for producing highly irregular-shaped parts. However, prior-evaluation by simulation before manufacture is also advised. Nevertheless, the complicated layer-by-layer printing process causes difficulties for traditional simulation methods and mesh generation methods. Recently, layer-based FEM methods have emerged to solve the difficulties and have been proved to be effective. However, the FEM have intrinsic disadvantages due to the needs of the element mesh. Especially for the 3D printing process simulation in which the deformation could be very large, this could deteriorate the accuracy due to excessive element distortion or even fail the simulation due to the element breakdown as mentioned in the introduction. On the other hand, the meshless method does not need element meshes; this becomes the main advantage to adopt meshless-based methods. Based on this idea, quite a few meshless methods have been developed and used in many engineering fields. However, few of them have been used for the 3D printing simulation, especially the simulation of the complicated layer-by-layer printing process. This is because the analysis model for an irregular-shaped object is complicated and the simulation process is different from normal structural problems. Most meshless methods are based on background meshes, even for commercial packages like LS-Dyna. Likewise, a layer-based model and a layer-by-layer analysis process are difficult for those which adopt the background mesh; hence, an effective method is needed for the meshless-based method. To solve the problems, this study, which is not based on the background mesh, proposed a series of geometry-related handling schemes to generate layer-based analysis models for irregular-shaped objects and proposed a step-by-step analysis process to simulate the layer-by-layer depositing process. The generation of the layer-based meshless model is straightforward and effective even for extremely irregular parts encountered in the biomedical field. With these, the 3D printing process for irregular-shaped objects can be simulated without difficulties. In addition, because the analysis model does not include any element mesh, the problems due to excessive element distortion can be avoided. The demonstrated cases and results proved its effectiveness.

## Figures and Tables

**Table 1:** Temperature history for each layer in the layer-by-layer simulation.

Lay no.	-	-	-	-	-	-	-	-
7	-	-	-	-	-	-	158.2→	54.4
6	-	-	-	-	-	158.2→	54.4→	53.5
5	-	-	-	-	158.2→	54.4→	53.5→	52.0
4	-	-	-	158.2→	54.4→	53.5→	52.0→	50.3
3	-	-	158.2→	54.4→	53.5→	52.0→	50.3→	48.6
2	-	158.2→	54.4→	53.5→	52.0→	50.3→	48.6→	47.1
1	158.2→	54.4→	53.5→	52.0→	50.3→	48.6→	47.1→	46.0

**Table 2:** Effective layer thickness for the real printing thickness of 0.1 mm.

Effective layer thickness (mm)	1.43	2.00	2.50	3.33	5.00	Exp.
Max. Disp. (mm)	3.16	3.66	2.3	1.42	1.27	3.31
Deviation (%)	4.53%	10.57%	30.51%	57.10%	61.63%	-

**Table 3:** Effective layer thickness for the real printing thickness of 0.2 mm.

Effective layer thickness (mm)	1.43	2.00	2.50	3.33	5.00	Exp
Max. Disp. (mm)	3.04	3.5	2	1.39	1.24	2.89
Deviation (%)	5.19%	21.11%	30.80%	51.90%	57.09%	-

**Table 4:** Effective layer thickness for the real printing thickness of 0.35 mm.

Effective layer thickness (mm)	1.43	2.00	2.50	3.33	5.00	Exp
Max. Disp. (mm)	2.85	2.91	1.71	1.47	0.86	2.62
Deviation (%)	8.78%	11.07%	34.73%	43.89%	67.18%	-

**Table 5:** Comparison between simulations and the real printing for hole cases.

Hole Type	Analysis by Finite Element Method (FEM) (mm)	Analysis by Meshless (mm)	Experiment (mm)	Deviation
Circular	3.91	4.01	3.76	6.6%
Rectangular	4.32	4.40	4.46	1.5%

**Table 6:** Effects of the radius.

Radius (mm)	Area of Hole (mm^2^)	Max. Disp. (Simulation) (mm)	Max. Disp. (Experiment) (mm)	Deviation
1	3.14	3.92	3.42	14.51%
2.5	19.64	4.01	3.76	6.77%
4	50.27	4.06	3.94	2.97%

**Table 7:** Effects of the position of the circular hole.

Radius (mm)	Position (from the Origin) (mm^2^)	Max. Disp. (Simulation) (mm)	Max. Disp. (Experiment) (mm)	Deviation
2.5	82.5	4.11	3.78	8.79%
2.5	87.5	4.01	3.76	6.77%
2.5	92.5	3.99	3.31	20.55%

**Table 8:** Maximum displacement comparison.

	Meshless Method (mm)	FEM (mm)	Deviation (%)
Maximum displacement	3.14	3.08	2%

**Table 9:** Comparison of the maximum displacements of the artificial joint.

	Meshless Method (mm)	FEM (mm)	Deviation (%)
Maximum displacement	3.752	3.793	1%

## Nomenclature

**α**	Thermal Expansion Coefficient
**B**	Strain-Displacement Matrix
**D**	Global Nodal Displacement Vector
d	Width of the Rectangular Hole (mm)
**ε**	Strain Vector
**ε** ^th^	Thermal Strain Vector
**E**	Constitutive (Material) Matrix
**F**	Global Loading Vector
**K**	Global Stiffness Matrix
**Ω**	Global Analysis Domain
**Ω**_i_	Domain of Layer i
r	Radius of the Circular Hole (mm)
T_m_	Melting Temperature (°C)
ΔT	Temperature difference (°C)
